# Association of apolipoprotein E genotype with outcome in hospitalized ischemic stroke patients

**DOI:** 10.1097/MD.0000000000008964

**Published:** 2017-12-15

**Authors:** Yajing Zhang, Shuling Liu, Wei Yue, Zhihong Shi, Yalin Guan, Mingzi Li, Yong Ji, Xin Li

**Affiliations:** a Department of Neurology, The Second Hospital of Tianjin Medical University,Tianjin, China; b Department of Neurology, Tianjin Huanhu Hospital, Tianjin, China; c Tianjin Key Laboratory of Cerebrovascular Disease and Neurodegenerative Disease, Tianjin Huanhu Hospital, Tianjin, China; d School of Nursing, Peking University,Beijing, China.

**Keywords:** apolipoprotein E, ischemic stroke, NIHSS, outcome

## Abstract

The aim of this study was to study the ability of the genotype to predict impairment and disability in hospitalized ischemic stroke (IS) patients after hospital discharge and 6 months after the onset of stroke symptoms.

A total of 786 patients with a first IS were enrolled. Apolipoprotein E (*ApoE*) polymorphism was examined using polymerase chain reaction. Stroke subtype was classified using the Oxfordshire Community Stroke Project classification scheme and the Trial of Org 10172 in Acute Stroke Treatment criteria. Impairment as assessed using the National Institutes of Health Stroke Scale (NIHSS), and disability as measured using the modified Rankin Scale (mRS), were compared against the *ApoE* genotype.

There was no significant association between the type of *ApoE* allele present and the stroke subtype. On multivariate regression analysis, the apolipoprotein E^∗^E4 allele genotype did not predict poor outcome at discharge and or at 6 months after stroke onset. A higher NIHSS score on admission, older age, and higher fasting glucose levels did predict poor outcome at hospital discharge. Higher glucose levels and higher NIHSS scores on admission were independent risk factors predicting poor neurologic status at 6 months after stroke onset.

The presence of the apolipoprotein E^∗^E4 and apolipoprotein E^∗^E2 genotypes, although related to cholesterol and triglyceride levels, do not affect recovery during rehabilitation. A higher NIHSS score on admission and a higher fasting glucose level predict poor neurologic status, both at hospital discharge and 6 months after onset.

## 1 Introduction

Ischemic stroke (IS) is an important cause of disability and death, with a high burden of disease seen in low-income and middle-income countries.^[1,2]^ The occurrence of IS is affected not only by risk factors such as hypertension, alcohol consumption, smoking, age, comorbidities, white blood cell count, blood glucose level,^[3]^ and cluster differentiation (CD) 28 null cells,^[4]^ but also by genetic factors such as apolipoprotein genetic polymorphisms. Among these, only apolipoprotein E (*ApoE*) polymorphisms have an important role in lipid transport and metabolism.

*ApoE* has 3 alleles—epsilon 2, epsilon 3, and epsilon 4—so it has 3 isoforms (E2, E3, E4). These, in turn, form 6 possible *ApoE* genotypes (E4/4, E3/4, E3/3, E2/4, E2/3, E2/2).^[5]^ The *ApoE* isoforms differ by amino-acid substitutions at positions 158 and 112. The order of prevalence is E3 >E4 >E2 in most populations.

*ApoE*^∗^E2 increases triglyceride (TG)and cholesterol levels, resulting in type III hyperlipoproteinemia.^[5]^*ApoE*^∗^E4 increases cholesterol, low-density lipoprotein (LDL), and apolipoprotein B *(ApoB)* levels, resulting in atherosclerosis, hyperlipidemia, and cardiovascular disease.^[5]^ The accumulation and over expression of *ApoE* can stimulate very-low-density lipoprotein (VLDL) and TG production, impair VLDL lipolysis, and cause hypertriglyceridemia. *ApoE* plays an important role in the normal metabolism of TG-rich lipoproteins: too much *ApoE* causes hypertriglyceridemia by impairing lipolysis and stimulating hepatic VLDL TG production, and too little *ApoE* impairs plasma clearance of TG-rich lipoproteins and their remnants.^[6]^

*ApoE*^∗^E4 increases the risk of cardiovascular disease.^[7]^ Some data suggest that *ApoE* is a regulator of bone metabolism and that *ApoE*^∗^E2 is a risk factor for low trabecular bone mass and vertebral fractures in humans.^[8]^*ApoE*^∗^E4 is the major known genetic risk factor for Alzheimer disease^[9]^; it increases the formation of amyloid plaques and neurofibrillary tangles^[10]^ and contributes to the pathogenesisof Alzheimer disease, especially the late-onset familial form.^[11]^ Moreover, patients with *ApoE*4 show an increased risk and an earlier onset of the disease.^[9]^*ApoE*^∗^E4 also affects the outcome of patients with acute head trauma^[12]^ and is associated with neurodegenerative disorders. It is suggested that *ApoE* is associated with the progression of amyotrophic lateral sclerosis^[13,14]^ and multiple sclerosis.^[15,16]^

Some researchers suggest that *ApoE* is related to the atherosclerotic process.^[17]^ This may be because atherosclerosis is both an inflammatory disease and a lipid disorder.^[18]^*ApoE* participates in lipid metabolism and accelerates atherogenesis. It has been shown that elevated levels of *ApoE* in plasma increase the risk of stroke.^[5]^ Some studies suggest that *ApoE*^∗^E4 is associated with a risk for IS^[19]^; however, the role of *ApoE* in IS remains debatable.^[20,21]^

We studied a total of 786 patients who were hospitalized with acute IS, with the aim of elucidating the relation between *ApoE* genotype and blood lipid levels, and of determining the predictive role of *ApoE* genotype in IS outcome and IS subtype in the Chinese population.

## 2 Materials and methods

### 2.1 Participants

A total of 786 consecutive patients with IS were recruited between December 1, 2014 and June 30, 2015 at Tianjin Huanhu Hospital, Tianjin, located in the northern part of China. The inclusion criteria were: a clinical diagnosis of acute IS at least 48 hours before; age 21 to 85 years; no previous stroke episodes; diagnosis confirmed by computed tomography (CT) or magnetic resonance imaging (MRI); patients with both small vessel occlusion and large artery atherosclerosis IS were included; all the patients were of Han ethnicity. Patients with a past history of dementia, primary subarachnoid hemorrhage, or previous IS were excluded, as were those with comorbidities including renal failure, valvular heart disease, or hepatic failure. Patients who used statin medications before the current attack were excluded. Patients with a National Institutes of Health Stroke Scale (NIHSS) ≥24 on admission (severe stroke) were excluded, as were patients who received intravenous thrombolysis, thrombectomy, or endarterectomy. We included patients who were treated with single antiplatelet agents and normal-dose statins. Doppler ultrasound of the neck vessels and transcranial Doppler were performed in all patients; when Doppler was not conclusive, CT or magnetic resonance angiography was performed.

Stroke subtype was classified as total or partial anterior circulation infarction, lacunar infarction, or posterior circulation infarction, according to Oxfordshire Community Stroke Project (OCSP) classification Scheme.^[22]^ Patients were classified as having large artery atherosclerosis or small vessel occlusionaccording to Trial of Org 10172 in Acute Stroke Treatment (TOAST) criteria.^[23]^ Patients who could not be subtyped according to either the OCSP or TOAST classification systems were excluded from the study.

Baseline information was collected within 8 hours of hospital admission by personal interviews with the patient or their family members. Demographic data, lifestyle risk factors, and medical history were collected using a standard questionnaire. Diabetes was defined as a fasting blood glucose level ≥7 mmol/L. Hypertension was defined as a systolic blood pressure ≥140 mmHg or a diastolic blood pressure ≥90 mmHg on 3 separate occasions.

### 2.2 Clinical assessment

Patients with acute IS were assessed at admission and at hospital discharge (3 weeks ± 3 days after onset) by experienced members of the medical staff, using the NIHSS and the modified Rankin Scale (mRS).Outcome was measured at hospital discharge using the mRS and the NIHSS. A score on mRS ≤2 and NIHSS ≤10 was classifiedas a “better outcome.”^[24]^

Patients were asked to return to the hospital 6 months after stroke onset for follow-up examination with mRS score determination. If a patient did not return, we telephoned and asked them about their condition, then recorded the mRS score.

### 2.3 Ethics statement

Written informed consent for study participation was obtained from each patient following a detailed explanation of the protocol and objectives.

### 2.4 Blood sample collection

Blood samples were obtained within 24 hours of admission, after patients had been in the supine position for 30 minutes and had fasted overnight for 12 to 14 hours. Peripheral venous blood from the antecubital vein was drawn from all patients. Blood samples were then placed into plasma separator tubes with K2-ethylenediaminetetraacetic acid and into serum-separator tubes for centrifugation.

### 2.5 Laboratory measurements

Total cholesterol (TC) and TG levels were measured using an enzymatic colorimetric method, and high-density lipoprotein cholesterol (HDL-C) was measured using the monophasic colorimetric method. Serum low-density lipoprotein cholesterol (LDL-C) was calculated usingthe Friedewald equation.^[25]^ Biochemical measurements were performed using a commercial kit (Shanghai Hua Chen Inc., Shanghai, China) and an automated analyzer (ADVIA2400; Siemens, Germany). Fasting glucose was measured using the glucose oxidase-perioxidase method.

### 2.6 Genetic analysis

Genomic DNA was extracted from whole-blood samples, and the *ApoE* gene was genotyped using polymerase chain reaction-restriction fragment-length polymorphism (PCR-RFLP) as described by Ji et al.^[26]^ A set of primers was designed to amplify the *ApoE* gene: 5′-TCCAAGGAG-GTGCAGGCGGCGCA-3′ (upstream) and 5′-ACAGAATTCGCCCCGGCCTGGTACACTGCCA-3′ (downstream). The PCR assays were carried out using 200 ng of genomic DNA, 25 pmol of primer, 2.5 μL of 10% dimethyl sulfoxide, and 0.5 U of *Thermus aquaticus* (Taq) DNA polymerase in a final volume of 25 μL. The DNA was denatured at 94°C for 5 minutes followed by 40 cycles of denaturation (94°C, 1 minute), annealing (65°C, 1 minute), and extension (72°C, 1 minute), with a final extension at 72°C for 10 minutes. The PCR product (20 μL) then was digested with 5 U of Cfo1 for at least 3 hours at 37°C, followed by electrophoresis for 2 hours at 200 V in 12% native polyacrylamide gel stained with 0.5 μg/mL of ethidium bromide. The DNA size was determined under ultraviolet light.

### 2.7 Statistical methods

Data were analyzed using the Statistical Package for the Social Sciences (version 16.0; SPSS Inc, Chicago, IL). The *χ*^2^ test and Fisher *F* test were used for independent categorical variables, and the Mann–Whitney *U* test and Student *t* test were applied for parametric and nonparametric variables, respectively. Data were expressed as the mean ± standard deviation or as the percentage, where appropriate. A *P* value of *<*0.05 was deemed to be statistically significant. A logistic regression model was used for multivariate analysis.

## 3 Results

A total of 786 patients (531 men and 255 women) with a mean age of 63.1 ± 11.6 years (range, 21–85 years) were hospitalized with their first IS during the study period. Owing to the very small number of patients with the E4/4 genotype (n = 7), the E3/4 and E4/4 genotypes were grouped together. There were no patients with the E2/2 genotype. Patients’ baseline characteristics according to *ApoE* phenotype are summarized in Table 1. Serum TG, TC, HDL-C, LDL-C, apolipoprotein A (*ApoA*), and *ApoB* levels were significantly different between *ApoE* epsilon (E2/3, E3/3, and E4) carriers. The patients with the E2/3 genotype had higher TG levels (*P* < .000), TC levels (*P* = .016), HDL-C levels (*P* = .007), and *ApoA* levels (P = .007), and lower LDL-C levels (*P* < .000) and *ApoB* levels (*P* < .000) than the patients with E3/3. The patients with E3/4 or E4/4 genotypes had higher LDL-C levels (*P* < .000) and *ApoB* levels (*P* = .001) than the patients with E3/3. There was no significant difference observed between the *ApoE* allele distribution and stroke subtype (Table 1).

**Table 1:**
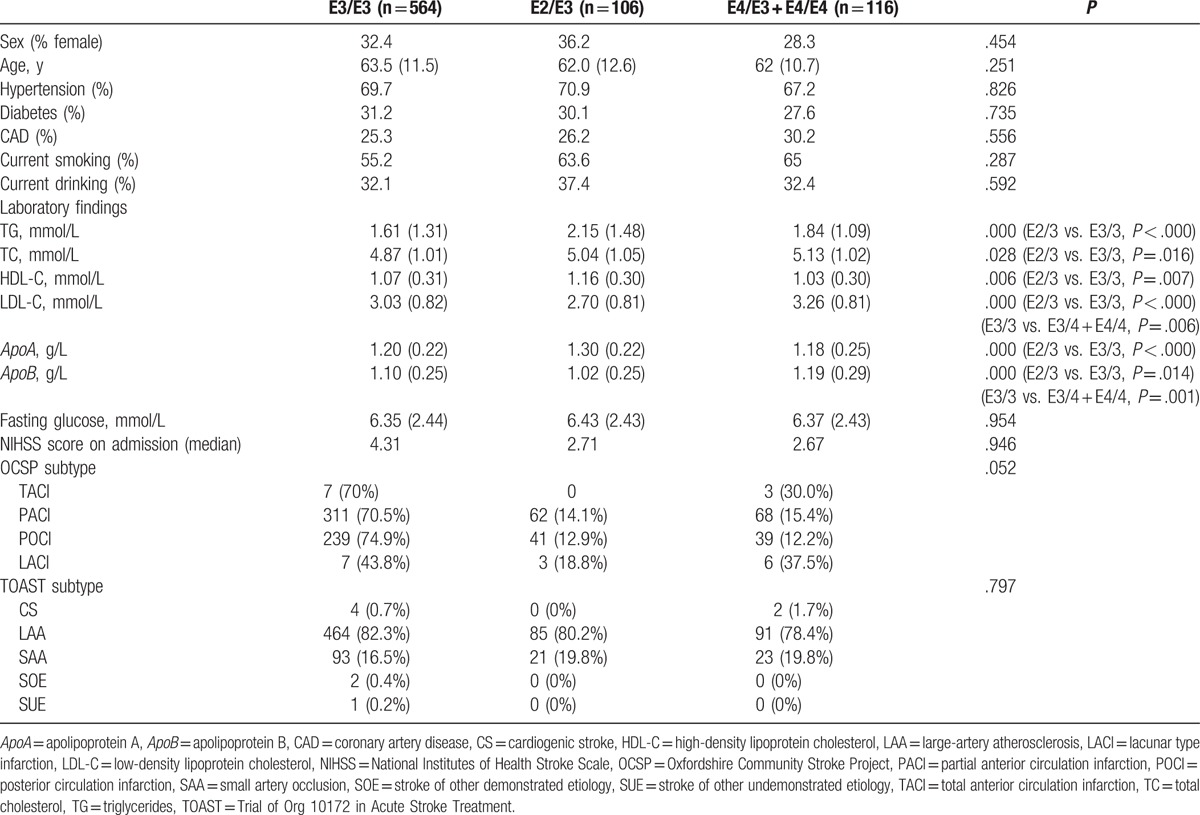
Baseline characteristics according to *ApoE* phenotype.

Univariate analysis using the mRS score at hospital discharge showed that the group with poor stroke outcome were of an older age and had higher TC levels, higher LDL-C levels, higher *ApoB* levels, and higher NIHSS scores on admission than those with a good stroke outcome (Table 2).On multivariate regression analysis, only older age and higher NIHSS scores on admission were independent risk factors predicting poor neurologic status at discharge (*P* < .000; Table 3).

**Table 2:**
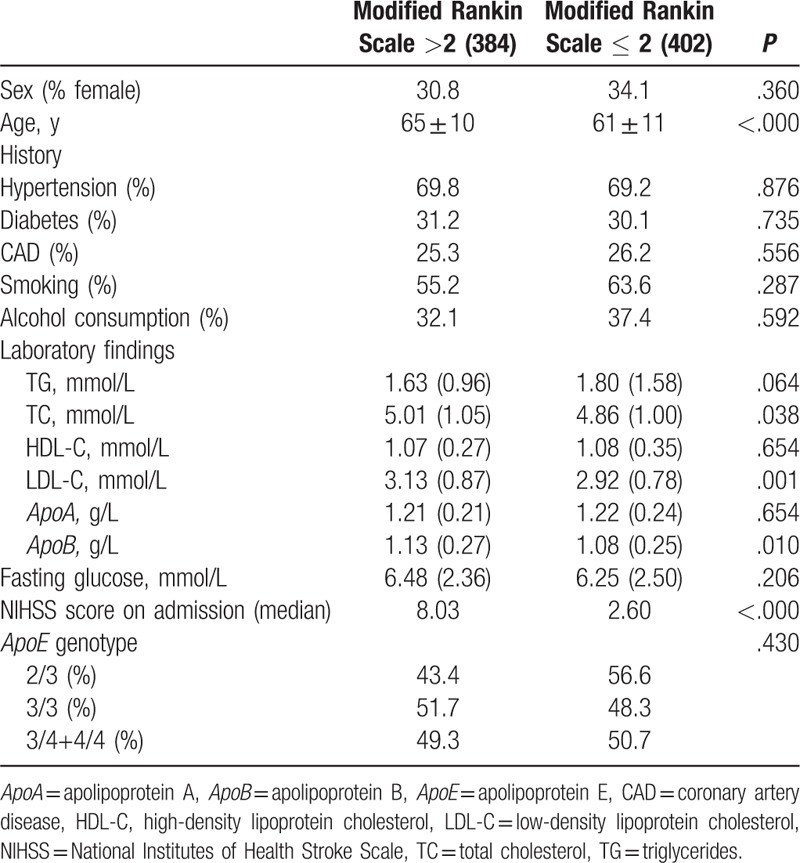
Sociodemographic and clinical features according to stroke outcome.

**Table 3:**
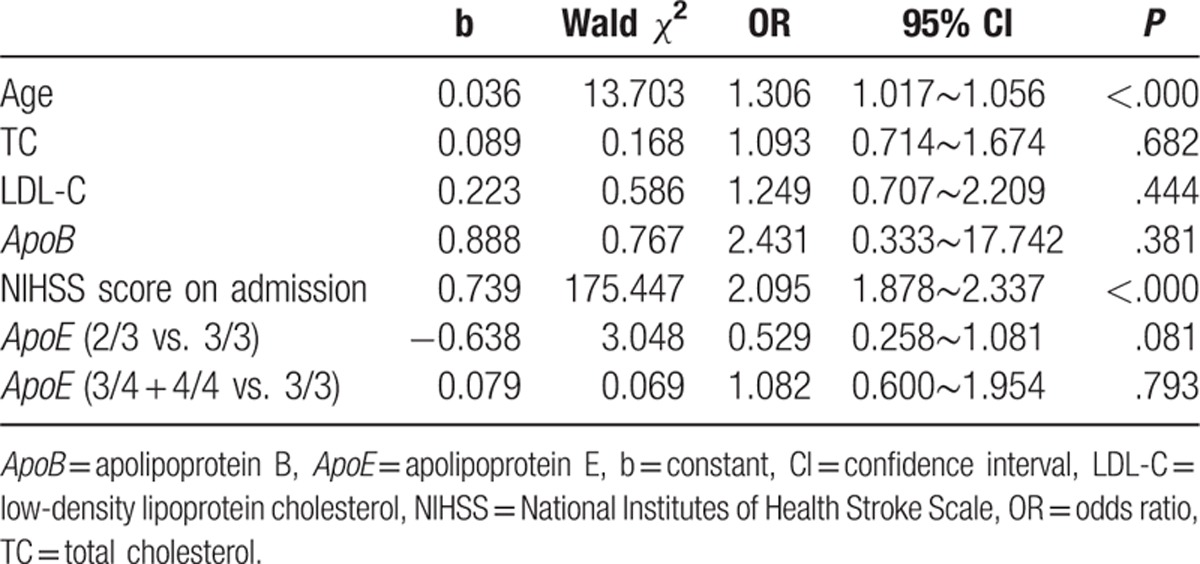
Factors influencing ischemic stroke.

On univariate analysis using the NIHSS score at hospital discharge, the group with poor stroke outcomes were of an older age and had lower TG levels, higher fasting glucose levels, and higher NIHSS scores on admission than those with good stroke outcomes (Table 4). On multivariate regression analysis, the *ApoE*^∗^E4 allele did not predict poor neurologic status at discharge. Older age, higher fasting glucose levels, and higher NIHSS scores on admission were independent factors predicting poor outcome at discharge (Table 5).

**Table 4:**
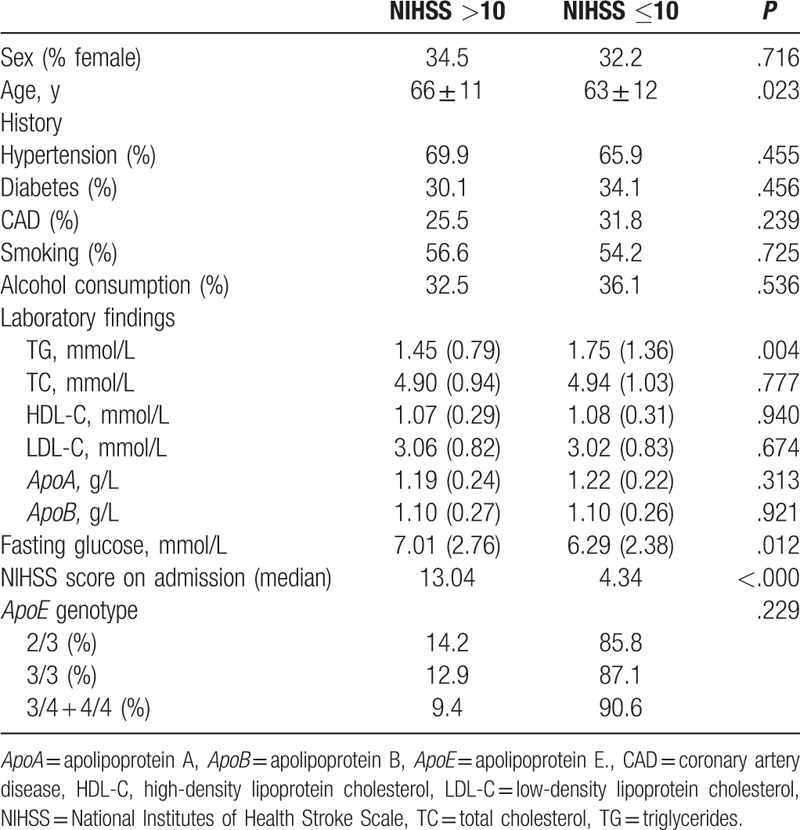
Sociodemographic and clinical features according to stroke outcome.

**Table 5:**
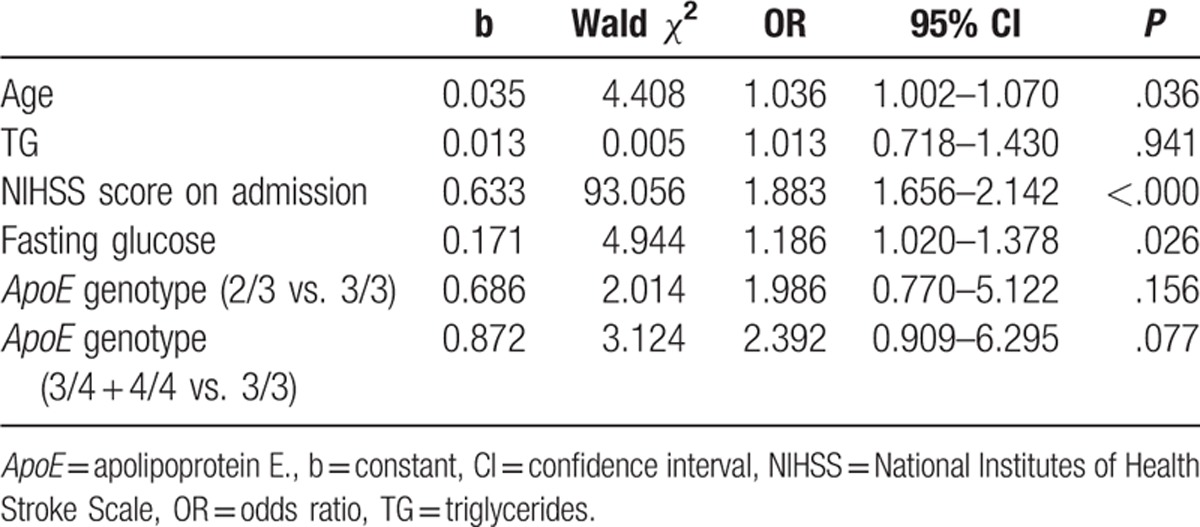
Outcome as measured by NIHSS score in patients with acute ischemic stroke.

On univariate analysis as measured by the mRS at 6 months after stroke onset, the group with poor stroke outcome had lower TG levels and higher NIHSS scores on admission than those with good stroke outcome (Table 6). On multivariate regression analysis, only higher blood glucose levels and higher NIHSS scores on admission were independent risk factors predicting poor neurologic status at 6 months after stroke onset (*P* = .015; *P* < .000; Table 7).

**Table 6:**
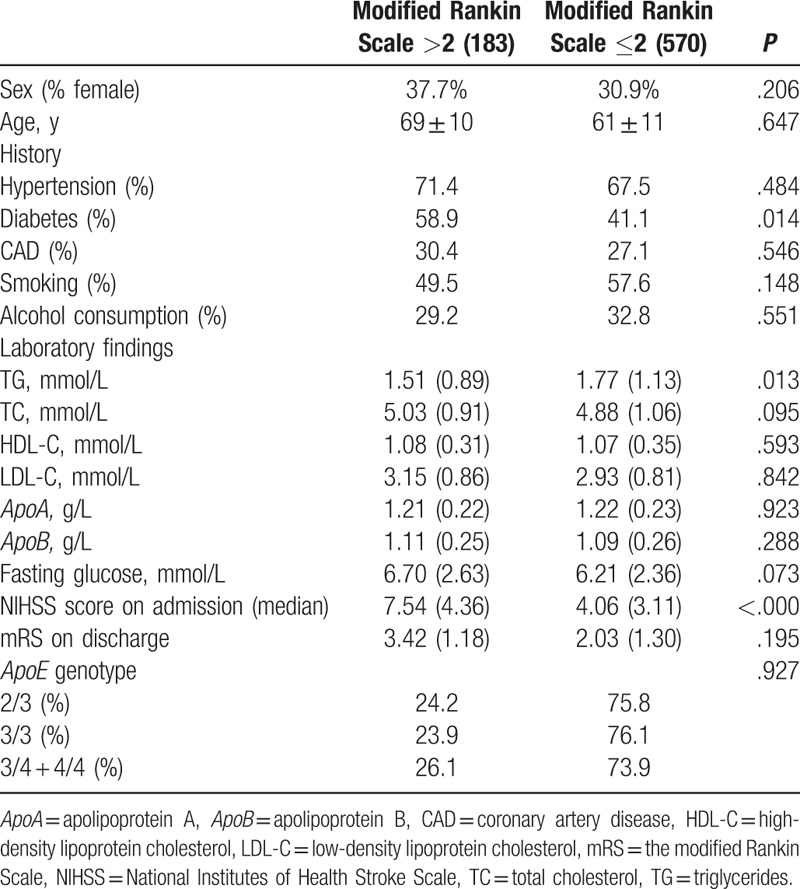
Sociodemographic and clinical features according to stroke outcome at 6 months after onset.

**Table 7:**
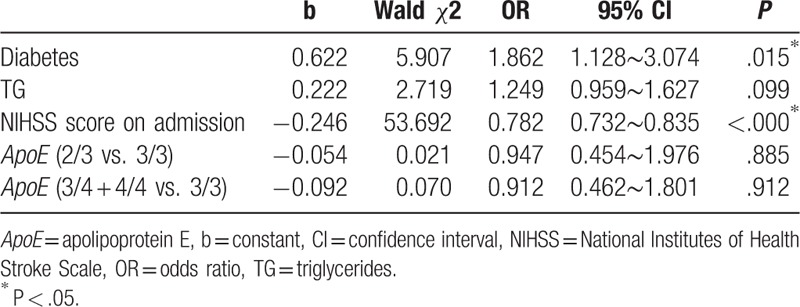
Factors influencing ischemic stroke at 6 months after onset.

## 4 Discussion

*ApoE*, a commonly investigated genetic marker, plays a central role in cholesterol and TG metabolism.^[27]^ The different *ApoE* alleles define different LDL-C levels: the lowest levels are seen with E2, the highest are seen with E4, and intermediate LDL-C levels are seen with E3. This variability appears constant in different populations,^[28]^ and our acute stroke patients were consistent with this pattern.

In our study of patients with acute IS, we found that the *ApoE* alleles exert an obvious effect on serum cholesterol and TG levels. Patients with E2/3 have higher TG levels, TC levels, HDL-C levels, and *ApoA* levels, and lower LDL-C and *ApoB* levels than patients with E3/3. Patients with E3/4 or E4/4 have higher LDL-C levels and *ApoB* levels than patients with E3/3.

The *ApoE*^∗^E4 allele has been shown to predict atherosclerosis and to be associated with the incidence of IS and hemorrhagic stroke.^[29,30]^ The E2 allele has also been shown to be associated with IS. McCarron et al showed that the *ApoE*^∗^E4 allele increases the risk of stroke.^[31]^ The *ApoE* allele has also been shown to predict functional outcome in survivors of traumatic brain injury.^[32–34]^ Alberts et al^[35]^ demonstrated that patients who survive intracerebral hemorrhage and who carry the *ApoE*^∗^E4 allele have poorer outcomes than patients who do not have the E4 allele. In other studies, however, the *ApoE* genotype has been shown to have no association with cerebral infarction.^[36]^ One study showed that the presence of the *ApoE*^∗^E4 genotype does not have a major influence on functional recovery after IS.^[37]^ Another recent study suggested that the *ApoE*^∗^E4 genotype increases the risk of cerebral infarction in the Chinese population.^[38]^ The possibility that the E4 allele of *ApoE* could act as a risk factor in IS is still controversial and remains a matter of ongoing study.^[36]^

Our *ApoE*^∗^E4 group had the highest LDL-C levels, and the *ApoE*^∗^E2 group had the lowest LDL-C levels, but *ApoE*^∗^E4 was not a dependent risk factor for IS. These results are in accordance with the “cholesterol paradox," wherein studies have confirmed that statins have a protective role in stroke prevention,^[39–41]^ yet the cholesterol level as a pathogenetic factor has not been confirmed in all trials.^[41,42]^ Some researchers have actually demonstrated an inverse relation between TC levels and cardiovascular disease. The mechanisms of the cholesterol paradox are not clearly understood, although inflammation may play a role. Reportedly, LDL-C levels in women are inversely related to levels of C-reactive protein.^[43]^ Therefore, a low LDL-C can enhance inflammation. In addition, higher LDL-C levels may reflect better health status overall and better tolerance of acute medical stress.

In this study, the short-term outcome after IS (measured by the NIHSS and the mRS at discharge) is related to patient age, NIHSS score on admission, and fasting glucose level, but not to plasma TC, LDL-C level, or *ApoE* genotype. These findings are consistent with those of other studies.^[44–46]^ Similarly, long-term outcome (mRS measured at 6 months after onset) is related to NIHSS score on admission and blood glucose levels, but not related to plasma TC, LDL-C levels, or *ApoE* genotype. It is possible that the NIHSS score on admission can indicate a patient's short-term and long-term outcome.

There are several risk factors for IS, including increasing age, hypertension, smoking, diabetes, race, and geographic location. This may explain the uncertainty surrounding the risk conferred by *ApoE*^∗^E4. We studied patients with IS and obtained results identical to those of some other researches. Our conclusion is that the *ApoE*^∗^E4 allele does not affect rehabilitation results after IS. However, further research, including long-term follow-up, is needed.

Our study is limited by the small sample size, the retrospective design, and the fact that the data were not broken down by age ranges. However, we have several advantages: we diagnosed and classified stroke subjects by serial CT or MRI, and patients were recruited in the acute phase of stroke, as early as possible within a 48-hour period after stroke onset.

In hospitalized patients with a first IS, we found that the presence of the *ApoE*^∗^E4 and *ApoE*^∗^E2 genotypes is related to cholesterol and TG levels, but does not affect recovery during rehabilitation. Older patient age, higher NIHSS scores on admission, and higher fasting glucose levels predict poor neurologic status at hospital discharge.
